# 

**Published:** 1888-07

**Authors:** 


CONTENTS:

ARTICLE I.—Mouth Microbes. By Dr. R. L. Cochran ..................... 97
" II.—Special Pathology of the Wisdom Teeth. By William Rushton ..................... 107
" III.—The Successful Transplanting of a Piece of Rabbit's Cornea into the Human Eye, for the Purpose of Restoring Sight to a Blind Man. By Prof. Julian J. Chisolm, M. D ..................... 118
" IV.—Empyema of the Antrum. By G. W. Watson ..................... 124
" V.—Resuscitation in Threatened Death from Chloroform. By Prof. F. T. Miles, M. D ..................... 129
" VI.—Painless Excavation by Anæsthetizing the Tooth with Ether Spray. By Dr. Ottolengui ..................... 131
" VII.—Devitalizing Pulps ..................... 133
Programme for the Joint Session of the American and Southern Dental Associations ..................... 135

EDITORIAL.

Dental Origin of Pyæmia ..................... 139

MONTHLY SUMMARY.

Blind Abscess ..................... 142

Destroying Pulps ..................... 143

Swaging Machine ..................... 144

